# Elucidation of *Akkermansia muciniphila* Probiotic Traits Driven by Mucin Depletion

**DOI:** 10.3389/fmicb.2019.01137

**Published:** 2019-05-22

**Authors:** Jongoh Shin, Jung-Ran Noh, Dong-Ho Chang, Yong-Hoon Kim, Myung Hee Kim, Eaum Seok Lee, Suhyung Cho, Bon Jeong Ku, Moon-Soo Rhee, Byoung-Chan Kim, Chul-Ho Lee, Byung-Kwan Cho

**Affiliations:** ^1^Department of Biological Sciences and KI for the BioCentury, Korea Advanced Institute of Science and Technology, Daejeon, South Korea; ^2^Laboratory Animal Resource Center, Korea Research Institute of Bioscience and Biotechnology, Daejeon, South Korea; ^3^Metabolic Regulation Research Center, Korea Research Institute of Bioscience and Biotechnology, Daejeon, South Korea; ^4^Infection and Immunity Research Laboratory, Metabolic Regulation Research Center, Korea Research Institute of Bioscience and Biotechnology, Daejeon, South Korea; ^5^Department of Internal Medicine, Chungnam National University School of Medicine, Daejeon, South Korea; ^6^Korean Collection for Type Cultures, Korea Research Institute of Bioscience and Biotechnology, Jeongeup-si, South Korea; ^7^Department of Bioprocess Engineering, Korea Research Institute of Bioscience and Biotechnology (KRIBB), School of Biotechnology, Korea University of Science and Technology, Daejeon, South Korea; ^8^114 Bioventure Center, HealthBiome, Inc., Daejeon, South Korea; ^9^Intelligent Synthetic Biology Center, Daejeon, South Korea

**Keywords:** *Akkermansia muciniphila*, extracellular protein, mucus layer, metabolic disorder, microbiome analysis

## Abstract

*Akkermansia muciniphila* is widely considered a next-generation beneficial microbe. This bacterium resides in the mucus layer of its host and regulates intestinal homeostasis and intestinal barrier integrity by affecting host signaling pathways. However, it remains unknown how the expression of genes encoding extracellular proteins is regulated in response to dynamic mucosal environments. In this study, we elucidated the effect of mucin on the gene expression and probiotic traits of *A. muciniphila*. Transcriptome analysis showed that the genes encoding most mucin-degrading enzymes were significantly upregulated in the presence of mucin. By contrast, most genes involved in glycolysis and energy metabolic pathways were upregulated under mucin-depleted conditions. Interestingly, the absence of mucin resulted in the upregulation of 79 genes encoding secreted protein candidates, including Amuc-1100 as well as members of major protein secretion systems. These transcript level changes were consistent with the fact that administration of *A. muciniphila* grown under mucin-depleted conditions to high-fat diet-induced diabetic mice reduced obesity and improved intestinal barrier integrity more efficiently than administration of *A. muciniphila* grown under mucin-containing conditions. In conclusion, mucin content in the growth medium plays a critical role in the improvement by *A. muciniphila* of high-fat diet-induced obesity, intestinal inflammation, and compromised intestinal barrier integrity related to a decrease in goblet cell density. Our findings suggest the depletion of animal-derived mucin in growth medium as a novel principle for the development of *A. muciniphila* for human therapeutics.

## Introduction

The gastrointestinal (GI) tract harbors complex and diverse microorganisms, termed the gut microbiota, establishing symbiotic interactions between the gut microbiota and the host (Hooper et al., 2012). In particular, the outer layer of the colonic mucus is densely populated by diverse commensal microbes (Johansson et al., 2011). Some of these gut microbes degrade mucin glycans via secretion of glycosyl hydrolases, and the resulting monosaccharides are used as additional energy and carbon sources. In turn, gut microbes also affect mucus composition by degrading mucin and releasing bioactive factors that can alter the gene expression of mucin-producing host cells (Deplancke and Gaskins, 2001; Johansson et al., 2011; Ottman et al., 2012). Due to these significant mucus–microbiota interactions, the mucus layer plays an essential role in shaping the mucus barrier and composition of the gut microbiota (Johansson et al., 2015). Recently, substantial evidence has demonstrated that the host–microbiota interaction has a critical role in obesity and other disorders by affecting several homeostatic (Cani et al., 2012; Everard et al., 2013), metabolic (Cani et al., 2007, 2008), and other physiological interactions with the hosts (Sommer and Bäckhed, 2013).

One of the key members of the colonic mucus-associated microbiota is *Akkermansia muciniphila*. This bacterium is capable of using mucus as a sole carbon and nitrogen source by producing several mucin-degrading enzymes (Derrien, 2004; Ottman et al., 2017a). It is well adapted to the mucous layer, accounting for 1–5% of the fecal microbial composition in healthy adults (Derrien, 2004; Belzer and de Vos, 2012). Thereby, *A. muciniphila* plays a crucial role in the maintenance of GI tract homeostasis and gut barrier integrity. Previous studies demonstrated that the abundance of *A. muciniphila* inversely correlated with several metabolic disorders (Dao et al., 2016; Derrien et al., 2017), such as obesity (Karlsson et al., 2012; Everard et al., 2013), inflammatory bowel disease (Png et al., 2010), type 2 diabetes (Zhang et al., 2013), and autism (Wang et al., 2011) in mice and humans. This correlation was additionally confirmed in several studies where oral administration of *A. muciniphila* bacteria reversed high-fat diet (HFD)-induced intestinal metabolic disorders and altered mucus layer thickness (Everard et al., 2013; Shin et al., 2014). Thus, *A. muciniphila* has garnered much attention as a next-generation probiotic bacterium (Cani and de Vos, 2017).

Among the critical factors that determine probiotic traits, extracellular proteins or vesicles are secreted into the host by probiotic bacteria. Some of these are reported to exhibit immunomodulatory and anti-inflammatory activity, with the secreted proteins potentially interacting directly with relevant immune cells to trigger downstream signaling pathways in the host mucosa (Sánchez et al., 2008, Sanchez et al., 2010; Bernardo et al., 2012; Ruiz et al., 2014). In this regard, the extracellular materials secreted by *A. muciniphila* have been evaluated in several studies. Remarkably, the cell-free supernatant of *A. muciniphila* culture was found to induce the production of an anti-inflammatory cytokine, interleukin-10, and this induction also occurred with the live bacterium (Ottman et al., 2017b), indicating that extracellular materials can activate the downstream signaling pathway of Toll-like receptor 2 (TLR2). A few studies have been conducted to determine the outer membrane proteome of *A. muciniphila* (Ottman et al., 2016, 2017b), leading to the discovery of the extracellular protein Amuc_1100, which recapitulates the effect of *A. muciniphila* on TLR2 activation as well as the improvement of intestinal barrier integrity (Plovier et al., 2016; Ottman et al., 2017b). Furthermore, it was revealed that *Akkermansia*-derived extracellular vesicles act as a functional module for maintaining the integrity of the intestinal barrier in HFD-mice (Chelakkot et al., 2018). However, it remains unknown how the expression of genes encoding extracellular proteins, including Amuc_1100, is regulated in response to dynamic mucosal environments. Such knowledge is imperative for better understanding the mechanisms of the interactions between the bacterium and the host as they relate to obesity, type 2 diabetes, and intestinal barrier integrity.

In the present study, we sought to elucidate the effect of mucin on the gene expression and probiotic traits of *A. muciniphila*. Transcriptome analysis revealed that the genes encoding most mucin-degrading enzymes were significantly upregulated in the presence of mucin [hereafter, mucin (+)]. By contrast, expression of most genes involved in glycolysis and energy metabolic pathways was upregulated in the absence of mucin [hereafter, mucin (-)]. In addition, 79 genes encoding secreted protein candidates, including Amuc_1100, were upregulated in mucin (-) conditions compared to mucin (+) conditions and the corresponding proteins secreted into culture medium. These changes in transcript levels were consistent with the fact that administration of *A. muciniphila* grown under mucin (-) conditions more efficiently reduced obesity and improved intestinal barrier integrity in HFD-induced diabetic mice than administration of *A. muciniphila* grown under mucin (+) conditions. These results suggest that the mucin content is important in the regulation of metabolism and gut permeability by *A. muciniphila*.

## Materials and Methods

### Bacterial Strain and Growth Conditions

*Akkermansia muciniphila* Muc^T^ (= DSM 22959^T^) was obtained from the German Collection of Microorganisms and Cell Cultures (Leibniz-Institut DSMZ-Deutsche Sammlung von Mikroorganismen und Zellkulturen GmbH, Germany). *A. muciniphila* was cultivated anaerobically at 37 °C on medium supplemented with mucin (DSM medium 1203a with 0.2% (wt/v) mucin) or without mucin^1^. DSM 1203a medium contained 16 g peptone, 7 g yeast extract, 5 g sodium chloride, 1 g starch, 1 g dextrose, 1 g sodium pyruvate, 1 g arginine, 0.5 g sodium succinate, 0.5 g L-cysteine HCl, 0.4 g sodium bicarbonate, 0.5 g ferric pyrophosphate, 0.005 g haemin, 0.0005 g vitamin K, and 0.5 g sodium thioglycollate in 1 l distilled water. All procedures for media preparation were performed under anaerobic condition according to previously established method (Ahn et al., 2016).

DSM 1203a agar plates (1.5%, w/v) were prepared and *A. muciniphila* DSM 22959^T^ was incubated at 37°C in the anaerobic chamber (Coy Laboratory Products) with a N_2_/CO_2_/H_2_ (86:7:7) gas phase.

### RNA Extraction and RNA-Seq Analysis

For RNA extraction, *A. muciniphila* grown on solid medium with/without mucin were harvested by scraping the surface using a sterilized scalpel and were resuspended in extraction buffer (200 mM Tris-HCl, pH 7.5, 25 mM EDTA, 250 mM NaCl, and 0.5% SDS). The harvested cells were rapidly frozen in liquid N_2_ and then ground to a fine powder using a mortar and pestle. Cell debris was removed by centrifugation at 3,000 × *g* for 30 min at 4°C. Total RNA was extracted using TRIzol^®^reagent (Thermo Scientific, Rockford, IL, United States) according to the manufacturer’s instructions. RNA quality was confirmed by the A260/280 ratio and visualization of two distinct bands of ribosomal RNAs (rRNA) using 2% agarose gel electrophoresis. rRNAs were selectively removed by Ribo-Zero^TM^ rRNA Removal Kit bacteria (Illumina, San Diego, CA, United States). RNA-Seq libraries were constructed in triplicate using TruSeq Strand mRNA LT Sample Prep Kit (Illumina) and then analyzed for size distribution using the Agilent Tapestation 2200 (Santa Clara, CA, United States). Constructed RNA-Seq libraries were sequenced with an Illumina Mi-Seq instrument with a 51 bp single-end sequencing recipe. The sequencing reads obtained were demultiplexed using Bcl2fastq V1.8.4 (Illumina), and low-quality reads and adaptor sequences were additionally trimmed using the CLC Genomics Workbench 6.5.1 (Qiagen, Hilden, Germany). The remaining reads were aligned to the *A. muciniphila* reference genome (CP001071.1) using CLC Genomics Workbench. The following parameters were applied for mapping: mismatch cost = 2, insertion cost = 3, deletion cost = 3, length fraction = 0.9, and similarity fraction = 0.9. The gene-wise read count was obtained using BEDTools suite v2.17.0 (Quinlan and Hall, 2010). Upon counting, reads that mapped only to the sense strand of the respective CDS were considered. Differential gene expression was assessed with the Bioconductor package DEseq2 (Love et al., 2014), and the significance level was set at *Padj* < 0.01. Total gene expression data can be found in Supplementary Table S2. Raw transcriptome data in FASTQ format is available in the European Nucleotide Archive (ENA) under study accession PRJEB28544.

### Annotation of Extracellular Proteins

All CDSs were analyzed using available prediction programs combined into a bioinformatics pipeline (Figure 3A) as described previously (Gomez et al., 2015; Cornejo-Granados et al., 2017). To predict signal peptide-carrying proteins, SignalP 4.1 (Dyrløv Bendtsen et al., 2004) was used with default parameters. Non-classical secreted proteins were additionally predicted with the options for “gram-positive bacteria” using SecretomeP 2.0 (Bendtsen et al., 2005), and all proteins with a SecP score > 0.5 were considered non-classical secreted proteins. Candidate secreted proteins derived from SignalP 4.1 and Secretome P 2.0 were merged and were additionally searched for homology to secreted protein by Blastp. LipoP 1.0 (Juncker et al., 2003) was used to predict lipoprotein motifs with default parameters, and all proteins annotated with “CYT (cytoplasmic)” were removed from the list of putative secreted proteins. Additionally, transmembrane proteins (with ≥ 1 transmembrane motifs) were removed from the set of predicted extracellular proteins using TMHMM 2.0 (Krogh et al., 2001).

### Animals and Diet

Eight-week-old male C57BL/6 mice were purchased from The Jackson Laboratory (Bar Harbor, ME, United States) and maintained at the Korea Research Institute of Bioscience and Biotechnology (Daejeon, Korea). Mice were housed under a constant 12 h light/dark cycle. To establish the obese mouse model, they were fed an HFD (60% of kcal from fat; D12492, Research Diet, New Brunswick, NJ, United States) for 6 weeks and then divided into three groups: HFD treated with 25% glycerol in sterile PBS (HFD group) or HFD treated with *A. muciniphila* grown on mucus-based [AK mucin (+)] or mucus-depleted [AK mucin (-)] medium. Mice were treated daily with an oral administration of *A. muciniphila* (1.0 × 10^8^ CFU/day) and treatment was continued for 4 weeks. Age-matched normal chow diet-fed mice were used as a control (ND group). Body weight and blood glucose were recorded once weekly. Oral gavage treatment with *A. muciniphila* was continued for 4 weeks. All animal experiments were approved by the Institutional Animal Care and Use Committee and performed in accordance with the institutional guidelines of the Korea Research Institute of Bioscience and Biotechnology (Approved No.: KRIBB-AEC-16117).

### IP-GTT and IP-ITT

After 4 weeks of HFD treatment with *A. muciniphila* administration, intraperitoneal glucose tolerance test (IP-GTT) and intraperitoneal insulin tolerance test (IP-ITT) were performed. After 16 or 4 h of fasting for GTT and ITT, respectively, the basal glucose level of each mouse was measured in blood taken from the tail vein. Then, either glucose (2 g/kg) or insulin (0.1 U/ml) was injected intraperitoneally, and blood glucose levels were monitored at 30, 60, 90, and 120 min. The area under the curve (AUC) was calculated using GraphPad Prism software (La Jolla, CA, United States).

### Tissue Sampling

At 4 weeks post-treatment, blood samples were collected from the orbital venous sinuses of the mice to analyze the concentrations of plasma insulin. Plasma was prepared by centrifugation at 10,000 × *g* for 5 min at 4°C. After exsanguination, mice were sacrificed by cervical dislocation. Liver, epididymal, and subcutaneous adipose and brown adipose tissues were collected for histology, and small intestine samples were immediately immersed in liquid nitrogen and stored at -80°C for further analysis.

### HOMA-IR

After 4 weeks of HFD treatment with *A. muciniphila* administration, plasma insulin levels were determined using an ELISA kit (Mercodia, Uppsala, Sweden) according to the manufacturer’s instructions. HOMA-IR was calculated using the following formula: fasting blood glucose (mg/dl) × fasting insulin (μU/ml)/405.

### Plasma LPS Analysis

Plasma LPS concentration was measured with a commercially available kit [Cambrex Limulus Amebocyte Lysate (LAL) kit; Lonza, Walkersville, MD]. This assay has a sensitivity range of 0.1–1.0 endotoxin units (EU)/ml. Plasma samples were diluted 1:5 in endotoxin-free water and then heated at 75°C for 5 min to remove interfering plasma components. Endotoxin assays were performed using a quantitative turbidimetric Limulus amebocyte lysate assay.

### Histological Analysis

At necropsy, liver samples and adipose tissues were immediately fixed in 10% formaldehyde, embedded in paraffin, and cut into 4 μm slices. Slides were stained with hematoxylin and eosin. Then, histology sections were viewed at 100× magnification, and images were obtained with a microscope (Olympus BX51, Tokyo, Japan). The total number and cross-sectional area of adipocytes were calculated with an image analysis program (ImageInside ver. 2.32, Olympus). For goblet cell staining, deparaffinized and rehydrated sections were stained with periodic acid-Schiff’s (PAS) reaction. The number of goblet cells per mouse ileum was counted in 10-well-oriented crypt-villus units.

### Quantitative Real-Time PCR

At necropsy, Peyer’s patches free part in ileum was collected and total RNA was prepared from this intestine samples using TRIzol reagent (Thermo Fisher Scientific). Quantification and integrity analysis of total RNA was performed by NanoDrop^TM^ spectrophotometer (Thermo Fisher Scientific). The cDNA was synthesized by reverse transcription, and real-time qPCR was performed as previously described (Kim et al., 2015). The 18S RNA gene was used as a reference. The sequences of the primers used for real-time qPCR are available in Supplementary Table S7.

### Statistical Analysis

Data were analyzed and statistical testing (Pearson’s correlation coefficient and Student’s *t*-test) was performed using GraphPad Prism v8 and R software. Comparisons multiple groups were performed using Tukey-Kramer HSD test after the one-way analysis of variance (ANOVA). The threshold of significance was set at *P* < 0.05. Replicates (*n*) in this study refer to biological replicates.

## Results

### Transcriptomic Landscape of *A. muciniphila* Under Mucin-Rich and -Depleted Conditions

**FIGURE 1:**
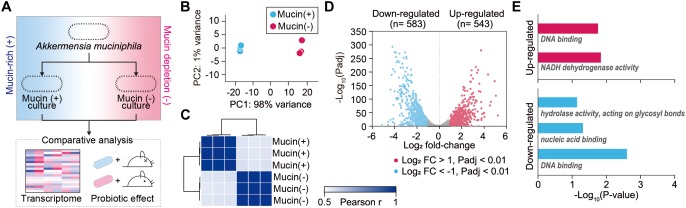
Transcriptomic dynamics of *A. muciniphila* under mucin-rich and mucin-depleted conditions. **(A)** Schematic of experimental design employed in this study. **(B)** Principal component analysis (PCA) of whole-transcriptome RNA data. **(C)** Sample-to-sample Pearson correlation coefficients between the data sets. **(D)** Volcano plot of genes sequenced under mucin-rich and mucin-depleted conditions. Differentially expressed genes (DEGs) are in blue. **(E)** Significantly enriched Gene Ontology (GO) molecular function terms among upregulated (red) and downregulated (blue) differentially expressed genes between mucin-rich and mucin-depleted conditions (Benjamini-Hochberg corrected *P* < 0.05).

To elucidate global changes in gene expression in response to the presence of mucin, RNA-sequencing (RNA-Seq) was employed for *A. muciniphila* cells grown under mucin (+) and mucin (-) conditions (Figure 1A). We obtained 1.9–3.8 million high-quality sequencing reads for each triplicate sample that mapped uniquely to the *A. muciniphila* reference genome (CP001071.1) with at least 36-fold sequencing coverage (Supplementary Table S1; van Passel et al., 2011). Principal component analysis of the RNA-Seq results showed significant changes in gene expression between the mucin (+) and (-) conditions, suggesting a global change in cellular functions (Figure 1B). Pearson correlation coefficient values between three biological replicates confirmed the experimental reproducibility (Pearson correlation coefficient > 0.99) (Figure 1C). Among the 1,126 DEGs between the mucin (+) and (-) conditions (Supplementary Table S2), 583 genes were downregulated and 543 genes were upregulated in mucin (-) conditions compared to mucin (+) conditions; the DEGs exhibited a considerable dynamic range of gene expression, with fold changes ranging from 0.03-to 41.01-fold (Figure 1D).

To gain more insight into the biological functions of the DEGs, Gene Ontology (GO) term enrichment analysis was conducted (Shannon et al., 2003; Saito et al., 2012). All enriched GO molecular function terms are shown in Figure 1E, with clear functional differences between up- and downregulated genes. GO terms enriched among upregulated genes included DNA binding (GO:0003677) and NADH dehydrogenase activity (GO:0003954) with Benjamini-Hochberg corrected *P* < 0.017. Furthermore, Kyoto Encyclopedia of Genes and Genomes (KEGG) pathway enrichment analysis showed that Ribosome and oxidative phosphorylation was significantly enriched, with a corrected *P* < 0.001 (Supplementary Table S3). Among the genes in this pathway, ATP synthase (Amuc_0505–Amuc_0510), the succinate dehydrogenase gene cluster (Amuc_0984–Amuc_0986), and the NADH dehydrogenase gene cluster (Amuc_1604–Amuc_1614) were significantly upregulated, with fold changes > 2.2 and *Padj* < 7.0 × 10^-219^. By contrast, enriched GO molecular function terms among the downregulated genes were significantly associated with hydrolase activity acting on glycosyl bonds (GO:0016798), nucleic acid binding (GO:0003676), and DNA binding (GO:0003677) with corrected *P* < 0.046 (Figure 1E and Supplementary Table S3). Considering that mucin is composed of heavily O-glycosylated glycoproteins, these results indicate that genes involved in mucin-degrading processes were overexpressed under mucin (+) conditions.

### Mucin-Rich Conditions Trigger Expression of Genes Encoding Mucin-Degrading Enzymes

**FIGURE 2:**
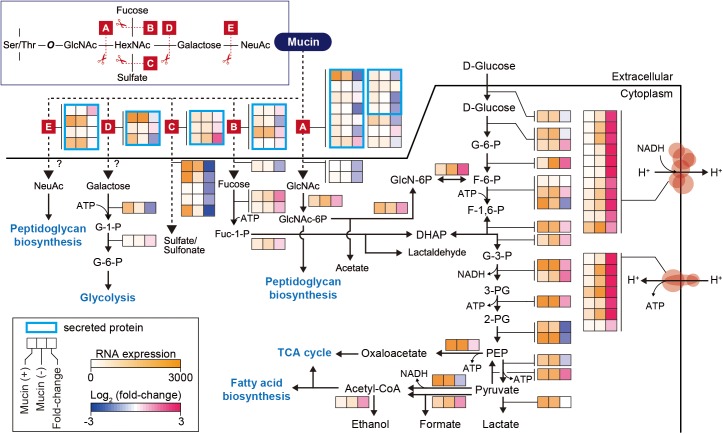
Differentially expressed genes involved in mucin-degradation pathway and glycolytic/gluconeogenesis in *A. muciniphila*. Enzymes are represented according to their corresponding mucin-degrading reactions, as follows: A, *N*-acetylgalactosaminidase; B, L-fucosidase; C, sulfatase; D, galactosidase; E, neuraminidase. The heat map indicates RNA expression levels and log_2_ fold changes. Gene list and expression values are also shown in Supplementary Table S4.

In further detail, we analyzed changes in the expression of genes involved in the mucin-degrading pathway and central carbon metabolism. Mucin oligosaccharides are composed of five different monosaccharides, including *N*-acetyl-D-glucosamine (GlcNAc), *N*-acetylhexosamine (HexNAc), L-fucose (fucose), D-galactose (galactose), and *N*-acetylneuraminic acid (NeuAc). Their heterogeneity is derived from various glycosidic bonds, and the sugar residue can be substituted with sulfate, acetate, or phosphate groups (Tailford et al., 2015). Therefore, the hypothetical mucin complex can typically be degraded by five different enzymatic reactions (A–E), shown in Figure 2. The corresponding enzymes are named according to their specific mucin-degrading reactions: *N*-acetylgalactosaminidases (A), L-fucosidases (B), sulfatases (C), galactosidases (D), and neuraminidases (E). For our analysis of the mucin-degrading pathway, we selected 28 enzymes that were predicted to be involved in mucin-degrading reactions by the BioCyc database (Caspi et al., 2016) and metabolic model (iAkkMuc_588) (Ottman et al., 2017a). Transcriptomic data showed that most genes encoding enzymes in the mucin-degrading A, B, and D groups were significantly downregulated under mucin (-) conditions, with fold changes < 0.79 and *Padj* < 0.01, indicating their crucial roles in mucin degradation (Figure 2 and Supplementary Table S4). Furthermore, most genes encoding mucin-degradation-associated transporters, such as fucose, GlcNAc, and sulfate transporters, were also significantly downregulated under mucin (-) conditions, with fold changes < 0.49 and *Padj* < 0.01. However, the transcript abundances of one gene encoding sulfatase in the mucin-degrading C group were highly upregulated under mucin (-) conditions, with fold changes > 3.76 and *Padj* < 0.01. These results are consistent with our previous understanding of transcriptomic and proteomic responses to mucin (Ottman et al., 2016, 2017a).

Additional reactions are necessary for converting monosaccharides into any of the intermediates of central carbon metabolism (Figure 2). These corresponding pathways were derived from the BioCyc and KEGG databases (Kanehisa, 2000; Caspi et al., 2016; Ottman et al., 2017a). Galactose is converted into glucose-6-phosphate (glucose-6-P) through the Leloir pathway, whereas GlcNAc is catabolized through glucosamine-6-phosphate (GlcN-6P) to fructose 6-phosphate (F-6-P). Fucose is catabolized through dihydroxyacetone phosphate (DHAP) to glyceraldehyde 3-phosphate (G-3-P) or through fuculose-1-phosphate to lactaldehyde. Additionally, NeuAc and GlcNAc can also be directly converted to peptidoglycan. Thus, the mucin-degrading pathway is also linked to the glycolysis pathway for catabolism. Interestingly, most of the genes involved in the glycolysis pathway showed similar expression levels between the two conditions, with *Padj* > 0.01, or significant upregulation under mucin (-) conditions, with fold changes of 1.4–3.5 and *Padj* < 0.01, with the exception of two enolase genes (Amuc_0844 and Amuc_1184), one dihydrolipoyl dehydrogenase gene (Amuc_1689), and one ATP-dependent 6-phosphofructokinase gene (Amuc_1481) (Figure 2 and Supplementary Table S4). In accordance with the upregulation of genes in the glycolysis pathway, respiratory chain complex genes were also significantly upregulated. As mentioned above, all genes encoding ATP synthase, succinate dehydrogenase, and NADH dehydrogenase were highly upregulated during growth without mucin (Figure 2 and Supplementary Table S4). Presumably, this regulation reflects a strategy to counteract the lower energy status in the absence of mucin, which has previously been shown to limit cell growth (Ottman et al., 2017a; van der Ark et al., 2018).

### Identification of Extracellular Proteins Encoded in the *A. muciniphila* Genome

**FIGURE 3:**
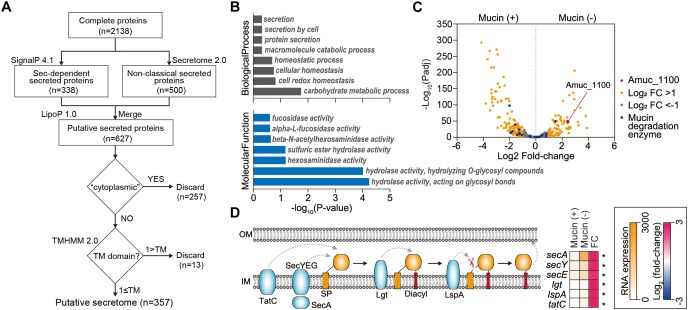
Analysis of extracellular proteins and protein secretion systems in *A. muciniphila*. **(A)** Workflow to identify the extracellular proteins encoded in *A. muciniphila* genome. **(B)** Significantly enriched Gene Ontology (GO) terms among the predicted extracellular proteins. GO terms belonging to the biological process and molecular function categories are shown in gray and blue, respectively. **(C)** Volcano plots summarizing the differentially expressed genes encoding extracellular proteins. The negative log_10_ of the *Padj* is plotted on the Y-axis, and the log_2_ of the fold change is plotted on the X-axis. **(D)** Differentially expressed genes involved in protein secretion pathways in *A. muciniphila*. ^∗^*Padj* < 0.01.

Next, we investigated extracellular or secreted proteins encoded in the *A. muciniphila* genome, as the extracellular materials of *A. muciniphila*, including the cell-free supernatant of *A. muciniphila* culture (Ottman et al., 2017b), purified Amuc_1100 protein (Plovier et al., 2016), and *A. muciniphila*-derived extracellular vesicles (Chelakkot et al., 2018), are able to recapitulate the effect of *A. muciniphila* in improving intestinal barrier integrity and the production of an anti-inflammatory cytokine. To identify genes encoding extracellular or secreted proteins, we set up a bioinformatics pipeline consisting of SignalP 4.1 (Dyrløv Bendtsen et al., 2004), SecretomeP 2.0 (Bendtsen et al., 2005), LipoP 1.0 (Juncker et al., 2003), TMHMM 2.0 (Krogh et al., 2001), and Phobious (Kall et al., 2007; Gomez et al., 2015; Cornejo-Granados et al., 2017; Figure 3A). These algorithms exhibited good performance in predicting the signal peptides, subcellular localization, and transmembrane helices of proteins. To predict classical and non-classical secreted proteins among the complete coding domain sequences (CDSs), SignalP 4.1 (Dyrløv Bendtsen et al., 2004) and SecretomeP 2.0 (Bendtsen et al., 2005) were used, respectively. Putative lipoprotein signal peptides were also identified using LipoP 1.0 (Juncker et al., 2003), and the predicted proteins were merged, resulting in a set of 627 unique proteins for *A. muciniphila* (Figure 3A). Among these, the presence of transmembrane regions was estimated by TMHMM 2.0 (Krogh et al., 2001), and proteins with no transmembrane domain were considered extracellular proteins. In total, the pipeline predicted 357 extracellular proteins (∼15.9% of the total proteins), which are listed in Supplementary Table S5. Amuc_1100, an outer membrane protein that has been shown to exhibit a probiotic effect on diet-induced obesity (Plovier et al., 2016), was also included among the extracellular proteins of *A. muciniphila*. Amuc_1100 was identified as a non-classical secreted protein without signal peptide type I, suggesting that this protein is not transported by the Sec proteins (Supplementary Table S5). In addition, the Amuc_1098 and Amuc_0336 proteins, which were found to be the most abundant outer membrane proteins through proteome analysis (Ottman et al., 2016, 2017b), were also classified as extracellular proteins by this analysis.

To understand the cellular functions of these extracellular proteins, we performed KEGG/GO term enrichment analyses for the 357 genes encoding these extracellular proteins. The results showed that 16 GO terms were significantly overrepresented in the biological process, molecular function, and cellular component categories, with *P* < 0.025 (Figure 3B and Supplementary Table S6). The most overrepresented GO term in the cellular component category was external encapsulating structure (GO:0030312), which is the representative cellular component reported for extracellular proteins (Gomez et al., 2015; Supplementary Table S6). Moreover, GO terms related to secretion, homeostasis, and carbohydrate metabolic process were highly enriched in the biological process category (Figure 3B). Interestingly, a significantly enriched term in the molecular function category was related to fucosidase, alpha-L-fucosidase, beta-*N*-acetylhexoaminidase, sulfuric ester hydrolase, hexosaminidase, hydrolase hydrolyzing O-glycosyl compounds, and hydrolase acting on glycosyl bonds, reflecting the mucin-degrading activity of the secretome (Figure 3B and Supplementary Table S6). This is consistent with the previously reported high fucosidase activity in the supernatant (Ottman et al., 2016). Furthermore, only the “other glycan degradation” KEGG pathway (00511) was significantly enriched, with a *P* < 4.3 × 10^-10^, indicating that mucin-degrading enzymes were highly enriched among the extracellular proteins. Among the 28 genes encoding mucin-degrading enzymes, 25 (89.3%) were predicted to be extracellular proteins (Figure 2). Taken together, the functions of the extracellular proteins are mainly related to mucin-degrading activity and tentative probiotic effectors (e.g., Amuc_1100).

### Mucin-Depletion Activates Expression of Genes Encoding 48 Secreted Protein Candidates and Protein Secretion Pathways

Next, we examined how the expression of genes encoding protein secretion systems and secreted proteins is affected by the presence of mucin. Using *Padj* and fold-change thresholds (*Padj* < 0.01 and |log_2_ fold-change| > 1), we identified a total of 197 DEGs, including 79 upregulated and 118 downregulated DEGs in mucin (-) conditions compared to mucin (+) conditions, that encoded extracellular proteins (Figure 3C). As mentioned above, genes encoding mucin-degrading enzymes were transcriptionally downregulated under mucin (-) conditions. By contrast, the gene encoding the Amuc_1100 protein was significantly upregulated (fold change = 5.40, *Padj* = 4.0 × 10^-44^) under the mucin (-) condition (Figure 3C), suggesting the enhancement of probiotic effects (Plovier et al., 2016).

To export these proteins from the cytosol into the host environment, bacteria use unique protein secretion systems. Although several protein secretion systems were not fully identified in the *A. muciniphila* genome, Sec, Tat (twin-arginine targeting), and a part of the type II secretion system were found in the genome. Interestingly, the mucin-depletion condition also significantly enhanced the expression of most genes in the Sec and Tat systems, as well as of those encoding lipoprotein localization-associated proteins (fold change > 2.6, *Padj* < 3.2 × 10^-18^) (Figure 3D). Taken together, the transcriptome analysis of *A. muciniphila* suggested that the presence or absence of mucin regulates the expression of Amuc-1100, as well as of the major protein secretion systems. These results indicate that the probiotic effect of *A. muciniphila* could be dependent on the mucin content of the culture conditions.

### Mucin-Depletion Enhances *A. muciniphila*-Mediated Effects on HFD-Induced Obesity

**FIGURE 4:**
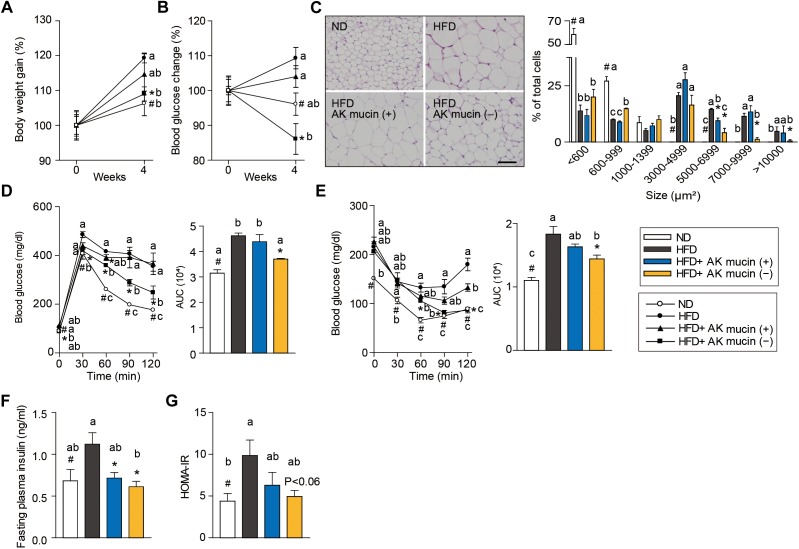
Effects of *A. muciniphila* on HFD-induced obesity. **(A)** Body weight and **(B)** blood glucose change (%) after 4 weeks of treatment. **(C)** Representative H&E-stained images and size distribution of epididymal adipose tissue deposits. Scale bars, 100 μm. **(D)** Blood glucose change and the mean area under the curve (AUC) measured during the IP-GTT. **(E)** Blood glucose change and the mean area under the curve (AUC) measured during the IP-ITT. **(F)** Fasting plasma insulin and **(G)** HOMA-IR index measured after 4 weeks of treatment. Data are means ± SEM (*n* = 4–5 for each group). ^#^*P* < 0.05, High fat diet (HFD) vs. Normal chow diet (ND) group, ^∗^*P* < 0.05, HFD vs. *A. muciniphila*-treated group (two-tailed Student’s *t*-test). ^abc^Means not sharing a common letter are significantly different at *P* < 0.05 (Tukey-Kramer HSD test).

In order to confirm the above hypothesis, we compared the effects of daily administration of *A. muciniphila* grown either on mucus-based [AK mucin (+)] or mucus-depleted [AK mucin (-)] medium on HFD-induced obesity. HFD-fed mice showed increased body weight (15%) and fasting blood glucose (12%) levels compared to control diet (ND)-fed mice after only 6 weeks of HFD treatment (Supplementary Figures S1A,B). These results indicate that HFD induces obesity and high blood glucose levels (Everard et al., 2013; Shin et al., 2014; Plovier et al., 2016). Interestingly, we found that an additional 4 weeks of treatment with AK mucin (-) attenuated HFD-induced changes in body weight and blood glucose levels more effectively than treatment with AK mucin (+) (Figure 4A,B).

The fact that treatment with *A. muciniphila* reduced body weight gain despite HFD consumption led us to additionally evaluate the effect of *A. muciniphila* on hepatic steatosis and adipocyte hypertrophy. Mice treated with AK mucin (-) exhibited a significantly lower hepatic triglyceride content (*P* < 0.05) than either untreated HFD-fed mice or HFD-fed mice treated with AK mucin (+) (Supplementary Figures S1C,D). Compared with the size distribution of epididymal adipocytes in the ND-fed group, small and medium adipocytes were significantly reduced (size > 1,000 μm^2^), but the large adipocyte fraction (size < 3,000 μm^2^) was significantly increased in HFD-fed mice (*P* < 0.05). The administration of *A. muciniphila* appeared to reduce adipocyte hypertrophy and increased the proportion of small adipocytes, which were more distinct in the HFD-fed mice treated with AK mucin (-) than in those treated with AK mucin (+) (Figure 4C). A similar trend was observed in subcutaneous adipocytes (Supplementary Figures S1E,F). Increased fat accumulation in white adipose tissue by various factors, including HFD treatment, has been shown to subsequently lead to ectopic fat deposition in brown adipose tissue (Calderon-Dominguez et al., 2016). When comparing mice treated with AK mucin (-) to HFD-fed mice, fat accumulation in brown adipose tissue was normalized (Supplementary Figure S1G).

To evaluate whether *A. muciniphila* had an effect on glucose intolerance and insulin resistance, intraperitoneal glucose tolerance test (IP-GTT) and insulin tolerance test (IP-ITT) were performed after 4 weeks of *A. muciniphila* treatment (Figure 4D,E). As expected, HFD-fed mice exhibited a significant increase in blood glucose levels (Figure 4D) and plasma insulin (Figure 4F) compared to ND-fed mice, both in the fasting state and during the glucose measurement, suggesting a decrease in glucose tolerance following HFD treatment. Moreover, HFD-fed mice treated with AK mucin (-) showed significant improvements in glucose tolerance and insulin sensitivity (*P* < 0.05), as evidenced by reductions in the area under the curve (AUC) and fasting plasma insulin levels compared to those in the untreated HFD-fed mice (Figure 4D–F). In addition, the homeostatic model assessment-insulin resistance (HOMA-IR) index, which is closely linked to insulin resistance status, was also significantly increased in HFD-fed mice. However, *A. muciniphila* treatment appeared to improve insulin resistance (Figure 4G). In particular, treatment with AK mucin ( - ) tend to improve insulin resistance than treatment with AK mucin (+). Although it was reported that there is no significant difference in probiotic effects based on the use of medium with or without mucin (Plovier et al., 2016), our results suggest that mucin-depletion conditions could enhance the beneficial effects of *A. muciniphila* compared to mucin-containing conditions.

### Mucin-Depletion Improves *A. muciniphila*-Mediated Effects on Intestinal Barrier Integrity and Inflammation

**FIGURE 5:**
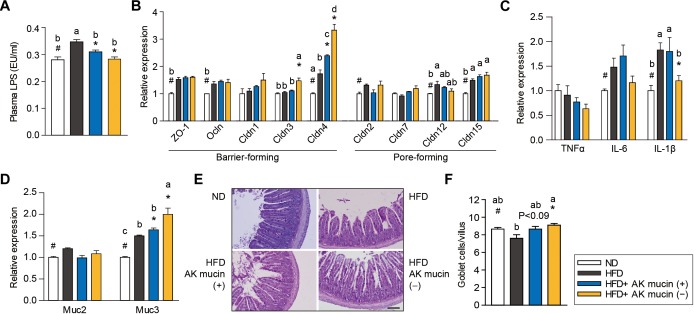
Effects of *A. muciniphila* on intestinal barrier function and inflammation. **(A)** Circulating plasma LPS measured after 4 weeks of treatment. **(B)** Expression of genes encoding barrier- or pore-forming tight junction proteins, **(C)** proinflammatory cytokines, and **(D)** mucin in the ileum. **(E)** Representative PAS-stained images and **(F)** mucus-containing goblet cell density in the ileum. Scale bars, 100 μm. Data are means ± SEM (*n* = 4–5 for each group). ^#^*P* < 0.05, High fat diet (HFD) vs. Normal chow diet (ND) group, ^∗^*P* < 0.05, HFD vs. *A. muciniphila*-treated group (two-tailed Student’s *t*-test). ^abc^Means not sharing a common letter are significantly different at *P* < 0.05 (Tukey-Kramer HSD test).

Altered gut barrier function is associated with increased intestinal permeability through decreased expression of tight junction proteins (Ahmad et al., 2014) in obese and diabetic mice. We therefore assessed the effects of AK mucin (+) or AK mucin (-) on endotoxemia and the expression of genes associated with the gut barrier. While HFD-fed mice displayed higher circulating LPS levels than ND-fed mice (Everard et al., 2013; Shin et al., 2014; Plovier et al., 2016; Chelakkot et al., 2018), suggesting metabolic endotoxemia, treatment with *A. muciniphila* restored LPS levels to that observed in the ND group, regardless of mucin growth conditions (Figure 5A). *A. muciniphila* treatment changed the expression of some genes encoding intestinal barrier proteins, as well as mucin. In the ileum, expression of genes encoding barrier-forming tight-junction proteins (CLDN3 and 4) was significantly higher in mice treated with AK mucin (-) (*P* < 0.05) than in untreated HFD-fed mice (Figure 5B). In addition, long-term HFD treatment has been shown to increase the expression of pro-inflammatory cytokines in the gut (Cani et al., 2008), and some cytokines can directly influence goblet cell function. Consistent with this, we observed that HFD treatment increased the expression of genes encoding pro-inflammatory cytokines (IL-6 and IL-1β); however, AK mucin (-) significantly inhibited intestinal inflammation (Figure 5C). Although the expression of *Muc2*, encoding a secreted form of mucin, was not altered, the expression of *Muc3*, encoding a membrane-bound mucin, was significantly upregulated by *A. muciniphila* treatment (Figure 5D). Moreover, treatment with AK mucin (-) led to greater goblet cell density in the ileum (Figure 5E,F). Therefore, this result suggests that in AK mucin (-)-fed mice, as compared to HFD-fed mice, more mucus is produced (McGuckin and Hasnain, 2017).

## Discussion

*A. muciniphila* is widely considered a next-generation beneficial microbe. This bacterium resides in the mucus layer of the host and regulates intestinal homeostasis and intestinal barrier integrity by affecting host signaling pathways (Dao et al., 2016; Cani and de Vos, 2017). Recently, several studies demonstrated that administration of *A. muciniphila* or *A. muciniphila*-derived compounds improves not only body weight and glucose tolerance, but also intestinal barrier integrity (Everard et al., 2013; Shin et al., 2014; Plovier et al., 2016; Chelakkot et al., 2018). Although the interaction between *A. muciniphila* and the mucus layer has been shown to be significant (Johansson et al., 2015), it remained unclear how this bacterium engages in crosstalk with host cells in the dynamic mucus layer environment.

In this study, we elucidated the effect of mucin on the gene expression and probiotic traits of *A. muciniphila*. Transcriptome analysis showed that most genes encoding mucin-degrading enzymes were significantly upregulated under mucin (+) conditions. By contrast, most glycolysis-related and energy metabolic pathway-related genes were upregulated under mucin (-) conditions. The observed changes in transcript levels reflect the fact that mucin-degradation-associated genes, as well as mucin-derived monosaccharide-associated transporters, were induced for cell growth with mucin, showing the adaptation of this gut bacterium to the mucosal niche (Ottman et al., 2017a; Thursby and Juge, 2017). However, the depletion of mucin induced a distinct response in terms of the upregulation of glycolysis and energy metabolic pathways, including NADH dehydrogenase and ATP synthase (Figure 2). In particular, the upregulation of these major genes related to energy metabolism indicates that *A. muciniphila* might be switching to preferred energy generation pathways under mucin (-) conditions. This regulation is presumably a strategy to adapt to the lower energy status under mucin-depleted conditions, which have been shown to limit cell growth (Ottman et al., 2017a).

Importantly, the absence of mucin induces expression of the gene encoding Amuc_1100. This result indicates that the beneficial effect of *A. muciniphila* may depend on the mucin content of the corresponding environment. In this context, the 78 upregulated extracellular proteins in mucin (-) conditions compared to mucin (+) conditions (fold change > 2, *Padj* < 0.01), similar to Amuc_1100, can be thought of as potential candidate proteins for beneficial effects of *A. muciniphila* in HFD-induced obesity, but additional confirmation is required. In addition, genes in the Sec and Tat secreted systems, as well as those encoding lipoprotein localization-associated proteins, were upregulated under mucin (-) conditions. Interestingly, prolipoprotein diacylglyceryl transferase (Amuc_1104) is located very close to the gene cluster (Amuc_1098–Amuc_1102) that includes Amuc_1100. Since various glycolipids and lipoproteins have also been shown to activate TLR2 in a similar manner to *A. muciniphila* (Yan et al., 2007), other lipoproteins besides Amuc-1100 may also be potential candidate proteins.

Several studies have highlighted the probiotic effects of *A. muciniphila* on host physiology, as mentioned above. As an extension of these studies, we demonstrated that the administration of *A. muciniphila* grown under mucin (-) conditions reduced obesity and improved intestinal barrier integrity in HFD-fed mice more efficiently than *A. muciniphila* grown under mucin (+) conditions. We observed that long-term HFD treatment without *A. muciniphila* resulted in reduced goblet cell density in the ileum, but in AK mucin (-)-fed mice, the reduced goblet cell density was restored. Based on these results, we speculate that the mucus layer of mice, which is thinned by obesity or diabetes (Everard et al., 2013; Shin et al., 2014), induces the expression of proteins capable of inducing a probiotic effect in *A. muciniphila*, resulting in the upregulation of the Muc3 gene and the regeneration of mucin, so that *A. muciniphila* is able to restore and colonize the mucus layer. This crosstalk between host cells and *A. muciniphila* is thought to be a strategy that allows the bacterium to survive in the intestinal mucus layer.

## Conclusion

In conclusion, this study revealed the effect of mucin on the gene expression and beneficial effects of *A. muciniphila* on HFD-induced obesity. The mucin content of the growth environment is crucial in inducing *A. muciniphila*-mediated improvements in the treatment of HFD-induced excessive body weight, glucose intolerance, intestinal inflammation, and compromised intestinal barrier integrity related to a decrease in goblet cell density. Our finding provides a novel principle for the development of *A. muciniphila* for human therapeutics and suggests avoiding animal-derived mucin in the growth medium for better probiotic activity of *A. muciniphila*.

## Author Contributions

B-CK, C-HL, and B-KC conceived and supervised the study. JS, B-CK, C-HL, and B-KC designed the experiments. JS, J-RN, D-HC, M-SR, and Y-HK performed the experiments. JS, SC, B-CK, C-HL, and B-KC analyzed the data. JS, SC, MK, EL, BK, B-CK, C-HL, and B-KC wrote the manuscript and commented on the manuscript.

## Conflict of Interest Statement

B-CK was employed by company HealthBiome, Inc. The remaining authors declare that the research was conducted in the absence of any commercial or financial relationships that could be construed as a potential conflict of interest.

## Footnotes

1 https://www.dsmz.de/microorganisms/medium/pdf/DSMZ_Medium1203a.pdf
