# Supplementary material for: Elucidation of Akkermansia muciniphila Probiotic Traits Driven by Mucin Depletion
Source: Front Microbiol. 2019 May 22;10:1137. doi: 10.3389/fmicb.2019.01137 (PMC6538878; doi:10.3389/fmicb.2019.01137)
Supplement: Supplementary file 8 [file Data_Sheet_1.pdf]

frontiers

Supplementary Material

Elucidation of *Akkermansia muciniphila* probiotic traits driven by mucin depletion

Jongoh Shin[1,#], Jung-Ran Noh[2,#], Dong-Ho Chang[3], Yong-Hoon Kim[2], Myung Hee Kim[4], Eaum Seok Lee[5], Suhyung Cho[1], Bon Jeong Ku[5], Moon-Soo Rhee[6], Byoung-Chan Kim[3,7,8,*], Chul-Ho Lee[2,*], and Byung-Kwan Cho[1,9,*]

*** Correspondence:** Byoung-Chan Kim, bckim@kribb.re.kr; Chul-Ho Lee, chullee@kribb.re.kr; Byung-Kwan Cho, bcho@kaist.ac.kr

Supplementary Figure

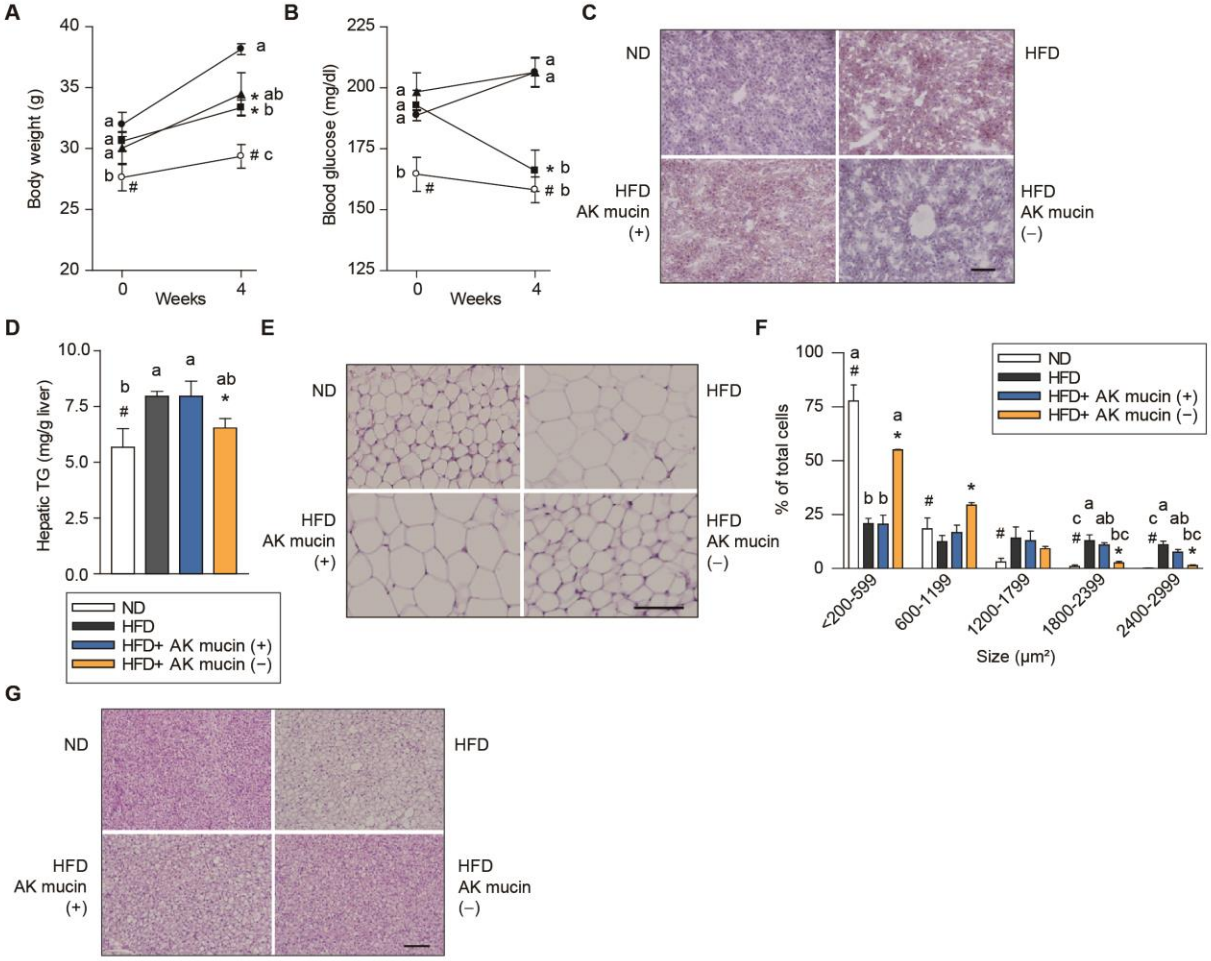

Supplementary Figure 1. Effects of *A. muciniphila* on HFD-induced obesity. (**A**) Absolute body weight (g) and (**B**) blood glucose (mg/dl) after 4 weeks of treatment. (**C**) Representative liver Oil-red O-stained pictures and (**D**) quantification of hepatic triglyceride content. Scale bars, 200 ㎛. (**E**) Representative H&E-stained pictures and (**F**) size distribution of subcutaneous adipose tissue deposits. Scale bars, 100 ㎛. (**G**) Representative H&E-stained pictures of brown adipose tissues. Scale bars, 100 ㎛. Data are means ± SEM (n=4-5 for each group). #*P*< 0.05, High fat diet (HFD) vs. Normal chow diet (ND) group, **P*<0.05, HFD vs. *A. muciniphila*-treated group (two-tailed Student's t-test). ^{abc}Means not sharing a common letter are significantly different at *P*<0.05 (Tukey-Kramer HSD test).
